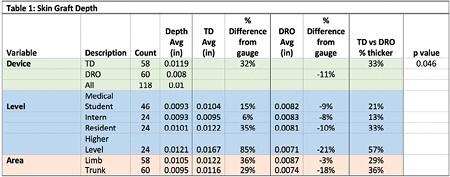# 113 A Study in Precision and Ergonomics: A Cadaveric Study

**DOI:** 10.1093/jbcr/irae036.112

**Published:** 2024-04-17

**Authors:** Genesy Aickareth, Elizabeth Brown, Alan Pang, John A Griswold, Debra J Kurtz

**Affiliations:** Texas Tech University Health Sciences Center, Lubbock, Texas; Texas Tech Univeristy Health Sciences Center School of Medicine, Lubbock, Texas; Kurtz Consulting Inc, vernon hills, Illinois; Texas Tech University Health Sciences Center, Lubbock, Texas; Texas Tech Univeristy Health Sciences Center School of Medicine, Lubbock, Texas; Kurtz Consulting Inc, vernon hills, Illinois; Texas Tech University Health Sciences Center, Lubbock, Texas; Texas Tech Univeristy Health Sciences Center School of Medicine, Lubbock, Texas; Kurtz Consulting Inc, vernon hills, Illinois; Texas Tech University Health Sciences Center, Lubbock, Texas; Texas Tech Univeristy Health Sciences Center School of Medicine, Lubbock, Texas; Kurtz Consulting Inc, vernon hills, Illinois; Texas Tech University Health Sciences Center, Lubbock, Texas; Texas Tech Univeristy Health Sciences Center School of Medicine, Lubbock, Texas; Kurtz Consulting Inc, vernon hills, Illinois

## Abstract

**Introduction:**

Split-thickness skin grafts have remained the mainstay treatment for 2nd and 3rd-degree burns. Traditional dermatome (TD) and dermatome with a rotating circular blade (DRO) are devices that have been used for skin grafting since the 1900s and 2013, respectively. Few studies have been conducted analyzing graft harvesting with the DRO. Our study analyzed skin depth consistency when harvesting with the DRO versus the TD. We conducted this study using varying levels of skin grafting expertise, gathered with both devices, and compared depth consistency across the graft.

**Methods:**

The study was conducted on a deidentified cadaver. There were 4 levels of harvesters: pre-clinical medical students, residents, a burn fellow, and a surgical technician with 10+ years of skin harvesting experience. All harvesters underwent basic training using each product’s instructional videos/training curriculum. The depth gauge of each device was set to 0.009 inches. Each subject harvested a 5-in length graft using each tool from the back and leg. The TD and DRO harvest occurred side-by-side at each location to minimize confounding. Each harvested 5-in section was punched in three locations diagonally with a 6 mm punch biopsy blade. Each punch was analyzed by dermatopathology for depth and sufficient graft harvest. The punches from each graft were analyzed for variance in depth against the gauge setting, and grafts from both devices were compared for depth consistency.

**Results:**

A total of 118 grafts were analyzed (Table 1). Grafts taken by the TD were significantly thicker than the DRO (p-value of <=.046). When compared to the depth gauge setting of 0.009 inches, the TD averaged 33% thicker and the DRO averaged 11% thinner. There were no significant differences between the levels of the harvesters nor the body location of the grafts.

**Conclusions:**

There are only a few studies that have been conducted comparing the DRO to the TD. In this study, the DRO was closer to the desired depth value than the TD regardless of skill level. This is significant because the participants had drastically varying familiarity with the tools themselves. This study was the medical student’s first time using the instruments, while the surgical technician used them for years. Additionally, the depth of the grafts taken by DRO was similar throughout all skill levels ranging from .0071-.0083. When using the DRO, all skill levels had decreased variability of thickness across the specimen. DRO is easier to use correctly as evidenced by the medical student and the resident who had similar depths of .0082 and .0081 respectively.

**Applicability of Research to Practice:**

This preliminary study shows that DRO offers skin grafts that are more consistent with the desired thickness and are uniform throughout the graft. A more constant graft thickness could possibly lead to better graft take and improved healing of the donor site.